# Response to Wright’s (2025) “Why There Are Exactly Two Sexes”

**DOI:** 10.1007/s10508-026-03418-0

**Published:** 2026-01-20

**Authors:** Dana Mahr

**Affiliations:** Institute for Technology Assessment and Systems Analysis, Karlsruhe Institute of Technology, Karlstraße 11, 76133 Karlsruhe, Germany

I write as a scholar of science and technology studies to critique Wright’s (2025) claim that biological sex is a priori “two-and only two” based on gamete type. This stance presumes a neutral, context-free scientific fact, but the science and technology studies show that even biology is filtered through social understandings. The very act of defining “male” and “female” by gametes is not a purely descriptive truth but a choice shaped by historical and cultural norms. As Jasanoff (2010) has argued, scientific knowledge is co-produced with social identities and institutions, meaning ways of knowing the world are inseparably linked to the ways people organize it. Wright’s “settled matter in modern biology” (that sex is strictly binary) is not free from this co-production. It reflects particular values about what counts as relevant evidence. Gieryn’s (1983) concept of “boundary-work” is instructive here: by insisting on a strict binary, Wright rhetorically draws a line around science that bolsters its authority at the expense of competing knowledge (such as gender studies and intersex biology). This ideological style serves scientific prestige, but it also silences alternative perspectives as if they were non-scientific.

Moreover, the rhetoric of pure “objectivity” hides subjective choices. Haraway’s (1988) notion of situated knowledge reminds us that claims of universal perspective are a god trick: an illusion of view-from-nowhere. Wright’s argument assumes science can transcend cultural context, but feminist epistemologists like Keller have shown that the history of science is gendered. Keller demonstrated how the demand for disembodied objectivity often masks biases that devalue traits coded as “feminine” (Vicedo, 2023). In this light, Wright’s exclusive emphasis on gametes ignores the many other biological features that vary (chromosomes, hormones, brain structures, etc.) and which social scientists have studied as interwoven with cultural sex/gender categories. Recent research in sexuality and gender studies highlights that the biological elements commonly linked to “sex” (including cells, tissues, molecules, structures, and pathways) do not neatly divide into two distinct categories (Bews & Marklein, 2025; Ritz, 2025). Framing sex strictly by gamete size is a reduction that omits these complexities, reflecting an a priori commitment to a binary rather than an exhaustive biological survey.

The implications of insisting on a single biological truth are deeply normative. Wright claims that definitional clarity offers “scientific and societal benefits,” but which society and whose science remain unstated. In practice, rigid essentialism about sex often undergirds exclusionary norms. For example, when policy-makers or courts cite “science” to restrict transgender rights, they use the same logic of a fixed binary that Wright espouses. Yet, as the interACT amici curiae brief observes, “sexual anatomy and physiology are not binary for many people,” and therefore “one’s ‘sex’ cannot be reduced to a straightforward function of body parts” (Supreme Court of the United States, 2019). By erasing this reality, Wright’s framing risks invalidating the lived experiences of intersex and gender-diverse individuals. It also overlooks the historical fact that assigning sex at birth is a subjective and culturally-mediated act (Supreme Court of the United States, 2019). An act that medical practices and laws have corrected only through social struggles (e.g., recognition of non-binary genders and intersex protections). As Fausto-Sterling (1993, 2025) has long emphasized and recently reiterated, rigid categories can obscure real variations: a large body of research shows that about 2% of people have intersex traits that challenge a simple XX/XY schema (Supreme Court of the United States, 2019). Ignoring this diversity in favor of a strict binary is not a neutral scientific outcome but a choice with ideological force.

STS teaches us to be wary of such scientific essentialism in policy and public discourse. History shows that when biology is treated as the sole arbiter of social categories, it often reinforces existing power structures. A few key risks include:

- Marginalization of diversity. A rigid binary provides “scientific” cover for excluding transgender and intersex people. It frames their identities as errors or delusions, rather than legitimate variations.
- Narrowing of inquiry. Focusing only on gamete-based definitions dismisses decades of scholarly work showing how sex and gender are entwined with culture (what Jasanoff calls the representations of science). It encourages cherry-picking of data that fit the binary narrative.
- False objectivity. This stance misuses the language of neutrality. As Haraway warns, a pretend neutrality (“god trick”) is not genuine truth but a politically-inflected position. Treating one view as the sole “objective” fact stifles pluralistic inquiry and ethical deliberation about what questions biology should address.

In sum, Wright’s portrayal of a hard binary sex ignores how scientific categories are historically and socially situated. Science is not insulated from culture; rather, how we define organisms (including humans) always involves interpretation. The co-production of knowledge and social order means that debates about sex and gender are as much about values and institutions as about cells and chromosomes. STS scholars like Haraway and Keller have shown that taking “nature” as self-evident often covers up contestable assumptions. In a time of vigorous discussion about gender diversity, invoking a single biological “truth” can shut down necessary conversations about intersex rights, health research, and inclusive policies.

Indeed, questions of definition are not purely academic. They shape how emerging technologies are designed, evaluated, and deployed: with material consequences for patient care and equity. For example, research on digital patient twins has demonstrated that model architectures and datasets that fail to integrate sex- and gender-relevant variables can systematically skew predictions and treatment suggestions, often privileging male-centric data while underrepresenting women and gender-diverse populations (Mahr et al., 2025; Weinberger et al., 2025). When digital twin models fail to account for menstrual cycles, sex-specific disease manifestations, or the lived realities of gender-diverse individuals (including, for example, the metabolic expressions associated with hormone replacement therapy) the consequences extend beyond abstract misclassification to real-world misdiagnosis, inappropriate clinical interventions, and the reinforcement of existing health inequities. Addressing these biases requires rethinking not only technical parameters but the epistemic commitments that undergird them; in other words, it demands reflection on how we define categories like “sex” before encoding them into predictive systems.

A more reflexive approach (one that acknowledges partial perspectives and the entanglement of biology with culture) is needed. Only then can scientific discourse on sex and gender avoid simply mirroring dominant ideologies under the guise of pure objectivity and instead contribute to technologies, policies, and clinical practices that are inclusive, equitable, and responsive to human diversity.
